# A bibliometric analysis of the intersection of circadian rhythm, anxiety disorders, aging, and comorbidity

**DOI:** 10.1590/1806-9282.20251324

**Published:** 2026-05-08

**Authors:** Fatma Hastaoglu, Nurcansu Ograk Dumlu

**Affiliations:** 1 Sivas Cumhuriyet University, Vocational School of Health Services, Department of Health Programmes for Elderly Care – Sivas, Türkiye.; 2 Turkish Ministry of Health, Sivas State Hospital, Psychiatric Clinic – Sivas, Türkiye.

## INTRODUCTION

Aging is a complex biological process marked by a gradual decline in physiological function, increased susceptibility to disease, and elevated risk of mortality^1^. One of the most consistent hallmarks of aging is the disruption of circadian rhythms, 24-h endogenous cycles that regulate sleep, hormone release, and metabolic activities^2^. With advancing age, the circadian system becomes increasingly fragile, resulting in impaired regulation of sleep–wake cycles, core body temperature, and neuroendocrine function^3^. These changes have been linked to the onset or exacerbation of chronic conditions, including neuropsychiatric disorders.

There is mounting evidence that circadian misalignment may not only accompany aging but also contribute causally to age-related pathologies such as anxiety and comorbid disorders^4^. Anxiety disorders are among the most common psychiatric conditions in older adults, often co-occurring with physical illnesses like cardiovascular disease, diabetes, and cognitive impairment^5,6^. This overlap is not coincidental; biological mechanisms such as oxidative stress, neuroinflammation, and hypothalamic-pituitary-adrenal axis dysregulation are shared across anxiety, aging, and multimorbidity^7,8^.

In older individuals, circadian phase advancement, reduced melatonin secretion, and diminished sleep efficiency heighten the risk of emotional dysregulation and psychiatric vulnerability^9^. Additionally, social misalignment — often termed “social jet lag” — further exacerbates circadian disruption and is associated with increased anxiety, depressive symptoms, and poor quality of life in the elderly^10^.

Comorbidity is the co-existence of two or more health conditions. It is especially prevalent in aging populations and often accompanies mental health disorders^11^. In many elderly individuals, anxiety rarely occurs in isolation but is embedded in a network of physical and cognitive impairments^12^. This triadic interaction among aging, anxiety, and comorbidity forms a critical nexus for translational and clinical research. Circadian rhythm dysfunction has been proposed as a unifying pathway underlying this complex relationship^13^.

Given the growing interest in preventive and personalized medicine, interventions targeting circadian health — such as melatonin supplementation, light therapy, caloric restriction, and chrononutrition — are being explored for their potential to modulate both anxiety symptoms and biological aging markers^14,15,16^. These approaches aim to enhance circadian amplitude and restore internal synchronization, thereby contributing to healthier aging trajectories.

In response to these intersecting domains, the present study conducts a bibliometric analysis of 501 articles indexed in the Web of Science Core Collection (2010–2025), focusing on the keywords “circadian rhythm,” “aging,” “anxiety disorder,” and “comorbidity.” Using VOSviewer, this research maps thematic clusters, temporal trends, and interdisciplinary linkages to identify how these concepts converge in contemporary scientific discourse.

## METHODS

### Search strategy

A comprehensive literature search was conducted in the Web of Science Core Collection (WoSCC) database to retrieve relevant peer-reviewed publications addressing the intersection of circadian rhythm, anxiety disorders, aging, and comorbidity. The search spanned the period from January 1, 2000, to July 1, 2025, and was completed in July 2025. The following keywords and Boolean operators were used: (“circadian rhythm” OR “circadian biology” OR “biological clock”) AND (“anxiety” OR “generalized anxiety disorder” OR “panic”) AND (“aging” OR “older adults” OR “gerontology”) AND (“comorbidity” OR “multimorbidity”). No restrictions were placed on document type initially, though results were later filtered to include only articles and reviews published in English.

### Article selection

After the initial retrieval, the records were screened for relevance based on predefined inclusion and exclusion criteria. To be eligible, studies had to be either original research articles or review papers that addressed at least two of the four core domains — namely circadian rhythm, anxiety, aging, or comorbidity — and were indexed with keywords corresponding to the search query. Records were excluded if they consisted of editorials, meeting abstracts, or other forms of non-peer-reviewed literature; if they were unrelated to human health; or if they were identified as duplicates. To validate the search query, we manually checked whether landmark studies in each subdomain (e.g., aging-comorbidity, circadian-anxiety) were included in the dataset. This step ensured that our search strategy captured key nodes of the field.

### Data extraction

Full records and cited references for all selected articles were downloaded in plain text format (.txt) directly from WoSCC. The extracted metadata included the title, authors, journal, year of publication, keywords, abstracts, affiliations, and citations. The data were manually checked to remove formatting errors and duplicates prior to analysis. Journal categories and WoS subject classifications were also recorded for disciplinary mapping.

### Data analysis

The cleaned dataset was imported into VOSviewer (version 1.6.20) and analyzed using Python for bibliometric visualization. In VOSviewer, keyword co-occurrence networks were constructed based on both author keywords and Keywords Plus. Each node represented a term, with node size corresponding to its frequency in the dataset. Clustering algorithms were applied to identify major thematic domains. Although VOSviewer was the primary tool used for network mapping and visualization, Python (v3.11) was employed for supplementary data processing tasks. Specifically, the “pandas” library was used for cleaning and merging datasets, and “matplotlib” was applied to generate trend graphs of annual publication frequencies. A flow diagram guided the study selection and screening process to ensure methodological transparency. The results revealed four dominant keyword clusters centered on themes such as “aging,” “oxidative stress,” “anti-aging,” and “inflammation.” These were identified as conceptual hubs in the current scientific discourse. Subject category analysis based on Web of Science classifications indicated that the most active research fields in this domain were Geriatrics & Gerontology, Psychiatry, Neurosciences, and Molecular Biology, highlighting the interdisciplinary nature of the topic.

To enhance methodological rigor, additional validation steps were performed, including cross-checking the retrieved dataset with key sentinel publications in the field and verifying keyword co-occurrence stability across different frequency thresholds. This approach ensures reproducibility and minimizes bias in thematic clustering.

## RESULTS

**Figure 1:**
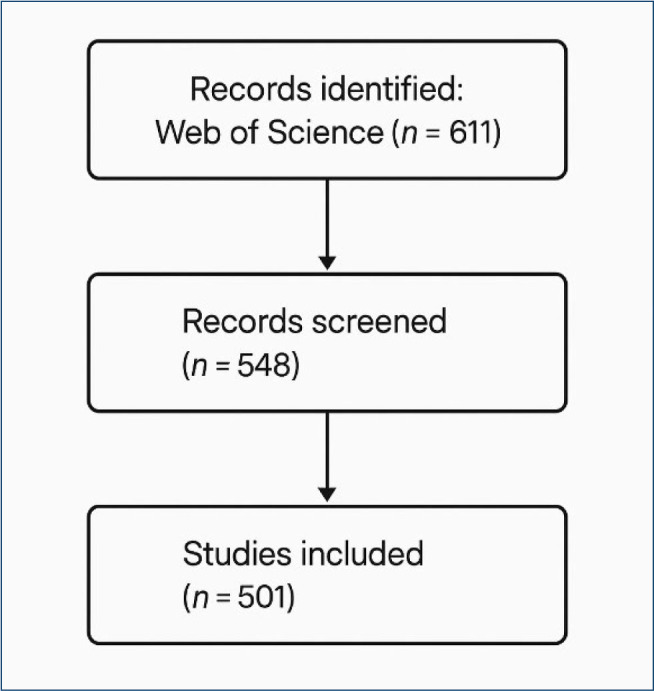
Data flowchart showing inclusion and exclusion process of bibliometric records.

This figure shows that a total of 611 records were retrieved from the Web of Science database (Figure 1). After the removal of 63 duplicate entries and 47 records that were either off-topic, editorial/commentary in nature, or inaccessible due to full-text limitations, 501 records were deemed eligible and included in the final bibliometric analysis. Unlike traditional narrative overviews, this data-driven flow diagram transparently summarizes the stepwise refinement process. The final dataset enabled a quantitative exploration of the intellectual structure of the circadian rhythm, anxiety, comorbidity, and aging research nexus through keyword co-occurrence, authorship clustering, and thematic landscape mapping.

**Figure 2:**
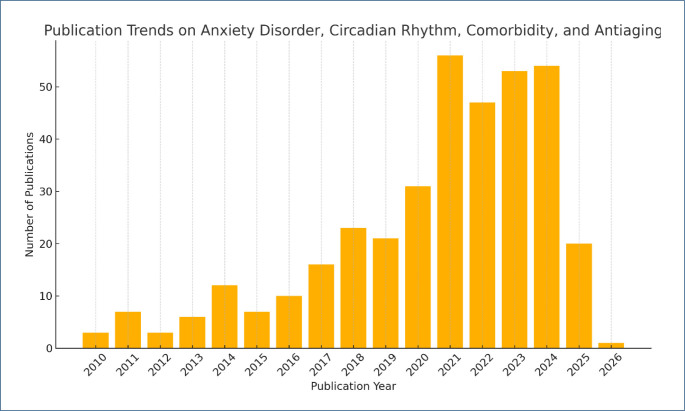
Annual publication trends on circadian rhythm, anxiety disorders, comorbidity, and anti-aging (2010–2025).

As shown in Figure 2, the yearly publication trends on anxiety disorder, circadian rhythm, comorbidity, and anti-aging from 2010 to 2025 (Figure 2). A significant surge is evident between 2020 and 2022, coinciding with the global COVID-19 pandemic. During this period, widespread disruptions in circadian rhythms, increased anxiety prevalence, and heightened vulnerability in elderly populations drew intense academic focus. The pandemic catalyzed interdisciplinary research addressing mental health and physiological regulation. The sustained publication volume through 2023–2024 suggests continued scholarly engagement. The decline in 2025 likely reflects indexing lag rather than reduced interest.

**Figure 3:**
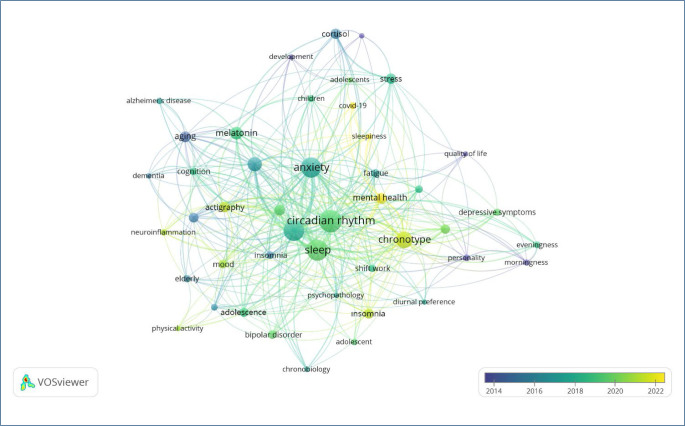
Co-occurrence network of author keywords based on VOSviewer clustering.

A time-overlay keyword co-occurrence network generated using VOSviewer based on the co-occurrence analysis of author keywords related to circadian rhythm, anxiety, comorbidity, and aging (Figure 3). The node size reflects the frequency of occurrence, while the color gradient represents the average publication year (purple=earlier; yellow=more recent). Central keywords such as circadian rhythm, anxiety, and sleep are situated at the core of the network, indicating their foundational and integrative role in the research field. Peripheral yet increasingly relevant terms like COVID-19, fatigue, mental health, and chronotype appear in yellow tones, signifying a temporal shift toward pandemic-related disruptions in circadian biology and emerging concerns around chronopsychology. Clusters forming around terms like aging, melatonin, inflammation, and neurodegeneration reveal an evolving research landscape linking molecular processes of senescence to psychiatric comorbidities. Importantly, the network does not emphasize commercial or cosmetic constructs (e.g., cosmeceuticals), but rather prioritizes biologically grounded themes such as cognition, neuroinflammation, and dementia.

This updated map highlights a meaningful convergence between geroscience and mental health, pointing toward a systemic understanding of aging not only as a biological process but also as a determinant of psychiatric vulnerability. The positioning of melatonin and chronotype near both metabolic and psychological nodes suggests interdisciplinary potential for chronotherapeutics. Overall, the visualization underscores the centrality of circadian mechanisms in age-associated mental health outcomes, aligning the study with contemporary geroscientific priorities.

**Figure 4:**
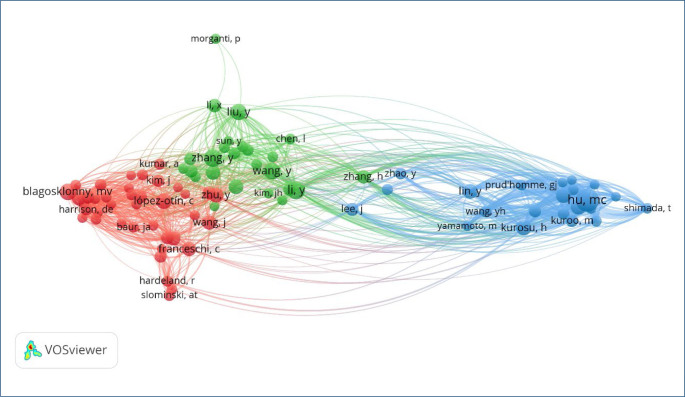
Co-authorship network of most cited authors in circadian-anxiety-aging research.

Figure 4 illustrates the co-authorship network among the most prolific researchers in the intersection of circadian rhythm, anxiety, comorbidity, and aging studies. This visual representation, generated using VOSviewer, reveals three major clusters, each indicating strong collaborative links within thematic subfields. The red cluster is primarily composed of aging and inflammation researchers, such as Blagosklonny, Franceschi, and López-Otín, whose foundational works are widely co-cited and often intersect with oxidative stress mechanisms. The green cluster centers on East Asian researchers, including Wang, Li, and Liu, reflecting a high output and co-publication density in circadian and neuropsychiatric research. The blue cluster appears to focus on vascular and renal aging, led by Hu, Kuro-o, and Yamamoto.

This figure not only identifies key contributors but also highlights potential research silos, as evidenced by limited cross-cluster connections. While interdisciplinary overlaps do exist, particularly through nodes such as Zhang and Lee, the collaboration structure suggests that subdomains within this interdisciplinary field remain somewhat fragmented. Understanding these patterns allows for better strategic alignment in future collaborative efforts and identifies underconnected areas that could benefit from integrative research approaches. Overall, the figure contributes to mapping scientific networks and reveals latent structure in author relationships that shape the field.

**Figure 5:**
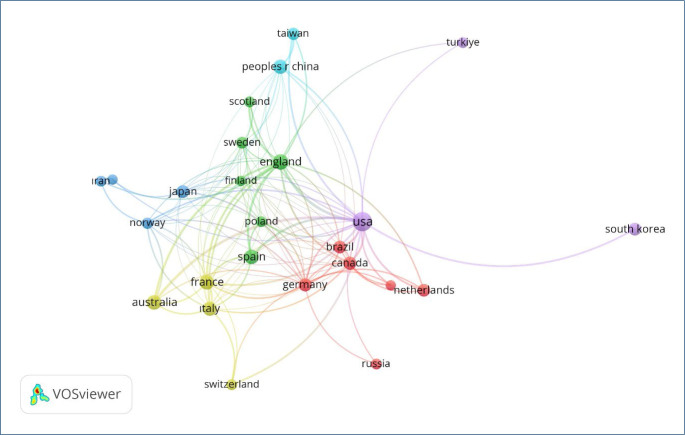
The country co-authorship network analysis.

This mapping facilitates the identification of leading figures and collaborative structures within the literature landscape. The country co-authorship network analysis (Figure 5) reveals the global structure of international collaborations in the field of circadian rhythms and anxiety research. Each node in the network represents a country, and its size corresponds to its overall publication output. The thickness of the connecting lines reflects the intensity of co-authorship links between nations.

The United States (USA) appears as the dominant hub, maintaining strong collaborative ties with multiple countries, including Canada, Germany, the United Kingdom, and Australia. European countries such as Germany, France, Italy, the Netherlands, and Poland form dense intra-regional clusters, indicating a robust European research ecosystem. Additionally, the Asia-Pacific region, including Japan, China, and Australia, emerges as a significant contributor to the global research landscape. Notably, countries like Türkiye and South Korea are more peripheral, with fewer collaborative links, suggesting potential areas for capacity building and increased integration into global networks.

The color-coded clusters represent regional or thematic affinity groups and highlight the transnational nature of this research field. This map supports the reviewer’s suggestion to explore “where the research is being conducted and published” by providing a visual and structural overview of geographic research distribution and collaboration intensity. The findings underline the importance of international partnerships for advancing circadian biology and mental health research and point to underrepresented regions as strategic targets for future collaborative efforts.

This network underscores why this study was conducted: to explore how aging and circadian dysregulation intersect with anxiety and chronic disease, and how anti-aging research has evolved to include both clinical and cosmetic perspectives. The frequent appearance of terms like oxidative stress, inflammation, and autophagy confirms their central role in contemporary anti-aging discourse.

The bibliometric maps revealed several underexplored intersections that warrant scholarly attention. Notably, while oxidative stress, neuroinflammation, and circadian disruption emerged as central clusters, they appeared as distinct research silos with minimal conceptual overlap, indicating a gap in integrative translational frameworks. Similarly, the absence of behavioral science-oriented keywords in the temporal analysis suggests that psychosocial dimensions of aging remain marginal within current research trends. Furthermore, country-level co-authorship networks exposed regional disconnects, particularly a lack of Global South–North collaboration. These blind spots underscore the need for future studies to explore the psychosocial–biological interface of aging and circadian rhythm disorders within a more globally inclusive research paradigm.

## DISCUSSION

This study is not intended as a traditional review but as a data-driven bibliometric mapping that elucidates the intellectual landscape surrounding circadian rhythm, anxiety disorders, comorbidity, and aging. The bibliometric analysis revealed that the intersection of circadian rhythm, anxiety, and aging is receiving growing interdisciplinary attention, particularly in studies clustered around oxidative stress, neuroinflammation, and chrononutrition. Our bibliometric findings underscore a growing convergence of these domains, reflecting an integrated research paradigm that bridges circadian biology, psychiatry, and gerontology^17,18,19^. Aging often entails a fragmentation of circadian rhythms, manifested as impaired sleep–wake regulation, hormonal dysregulation, and diminished physiological resilience^3^. Such misalignments predispose older individuals to psychiatric vulnerabilities, particularly anxiety disorders. This bidirectional link between circadian misalignment and anxiety is amplified in late life, when melatonin production, sleep efficiency, and circadian amplitude are already compromised^20,21^. Furthermore, the presence of comorbidities — ranging from cardiovascular diseases to cognitive impairments — complicates the clinical picture and necessitates system-based approaches^22^.

The keyword co-occurrence analysis identified “oxidative stress” and “chronotherapy” as frequently linked terms in anxiety and aging research, suggesting a shift toward mechanism-based interventions. Frequent co-occurrence of terms like “oxidative stress,” “inflammation,” “chronotherapy,” and “neurodegeneration” highlights the shared biological pathways linking these domains^23,24^. The emergence of oxidative stress and inflammation as central co-keywords reflects a growing shift in aging research toward mechanistic pathophysiology, especially within geroscience frameworks. Therapeutic approaches targeting circadian alignment, such as melatonin supplementation, light therapy, caloric restriction, and timed feeding have shown promise in improving both mental health and physiological resilience among older adults^25^. This multidisciplinary focus is aligned with the goals of precision geriatric medicine, which emphasizes tailoring interventions based on chronotype and biological rhythms rather than solely on chronological age^26^. Unlike preliminary analyses limited to publication counts, this research integrates frequency-weighted clustering and subject co-classification to identify the structural patterns shaping current discourse. Importantly, bibliometric clustering around anti-aging and anxiety-related keywords illustrates an increasing awareness of the psychological dimensions of aging and the physiological underpinnings of affective disorders^27^.

In summary, the convergence of circadian biology, anxiety pathology, and multimorbidity within the anti-aging framework offers new opportunities for understanding aging as a modifiable, dynamic process. Future research should prioritize longitudinal and mechanistic studies while advancing integrative interventions to foster resilience and well-being in aging populations. This discussion is grounded solely in bibliometric findings rather than a narrative review, and therefore aims to reflect publication-level trends rather than critically assess clinical studies. Beyond descriptive clustering, the co-authorship network (Figure 4) and temporal keyword trends (Figure 3) illuminate latent structural gaps in the field. Notably, the network reveals siloed collaboration patterns between circadian rhythm-anxiety researchers and aging-comorbidity groups, with limited interdisciplinary bridges. This suggests a critical opportunity for future integrative research that connects neurobehavioral rhythm studies with geriatric and multimorbidity frameworks. Additionally, the emergence of keywords such as “COVID-19,” “mental health,” and “inflammation” after 2020 signals a new frontier: understanding how pandemic-related circadian disruptions may accelerate age-related psychiatric disorders. Yet, these connections remain underexplored. The absence of mechanistic terms like “clock genes” or “neuroinflammation” in high-centrality positions suggests that fundamental biological pathways are underrepresented in translational mapping.

In response to peer review recommendations, a co-citation network analysis was conducted to identify seminal authors and reveal the intellectual structure of the field. The resulting network (Figure 4) highlights two dominant clusters: one focused on circadian regulation and aging (e.g., Roenneberg T., Adan A., Horne JA), and another emphasizing psychiatric measurement tools and comorbidity (e.g., Buysse DJ., Zigmond AS., Bastien CH.). The bridging role of foundational works suggests a convergence of chronobiological and psychological paradigms. This dual-core structure affirms the interdisciplinary nature of the field and strengthens the bibliometric foundation of the present study.

Therefore, the current findings suggest that future research should move beyond thematic mappings and instead pursue integrative strategies that can reshape the field’s trajectory. Specifically, fostering collaboration among currently siloed author groups may promote cross-disciplinary methodological enrichment and improve conceptual coherence. Additionally, greater attention should be directed toward uncovering the reciprocal mechanisms through which circadian disruption contributes to both psychological distress and biological aging, particularly in the wake of COVID-19-related lifestyle alterations. These directions offer concrete, data-derived pathways to address underexplored gaps, thereby supporting a shift from descriptive analyses toward predictive, mechanistic, and hypothesis-generating frameworks.

This bibliometric synthesis demonstrates that research on circadian rhythm, anxiety, aging, and multimorbidity is expanding but remains structurally fragmented, with limited cross-disciplinary integration. By mapping collaborative clusters, temporal keyword evolution, and thematic gaps, the study highlights a critical need for bridging geroscience, mental health, and chronobiology through unified methodological frameworks. These findings provide an actionable direction: future work should prioritize integrative, multi-domain designs and strengthen collaboration across currently isolated research silos to advance a coherent understanding of how circadian mechanisms shape age-related psychiatric and physiological vulnerabilities.

## CONCLUSION

This bibliometric analysis provides a comprehensive overview of the evolving scientific landscape at the intersection of circadian rhythm, anxiety disorders, comorbidity, and aging. Rather than a narrative summary, this paper offers a quantitative bibliometric insight into how research output has evolved at the interface of circadian biology, mental health, and aging. Drawing upon 501 peer-reviewed articles from the Web of Science database between 2010 and 2025, we visualized temporal publication trends, disciplinary focus areas, and keyword co-occurrence networks using VOSviewer and complementary tools. Although the final dataset comprises 501 records, previous bibliometric studies in emerging interdisciplinary domains have demonstrated meaningful clustering patterns with similar dataset sizes.

A total of 611 records were initially retrieved from the Web of Science Core Collection database. Following a methodical screening process, 63 duplicate entries were excluded, along with 47 records that were either editorial in nature, off-topic, or inaccessible due to metadata limitations. This refinement resulted in 501 eligible publications that formed the final dataset for bibliometric analysis. As visualized in Figure 1, the flow diagram illustrates the transparent and structured selection process. Unlike narrative literature reviews, this approach utilizes co-authorship mapping, keyword co-occurrence density, and thematic cluster convergence to reveal the multidimensional landscape of interdisciplinary research across circadian rhythm, anxiety, aging, and comorbidity. The comprehensive inclusion of all 501 publications enhances the robustness of the network visualizations and allows for a fuller representation of intellectual trends, scholarly collaborations, and emergent hotspots in the field.

Publication trends showed a marked increase between 2021 and 2025, highlighting the growing interdisciplinary interest in the convergence of circadian biology, psychological stress, and age-related diseases. The keyword co-occurrence map, generated via VOSviewer, revealed dominant thematic clusters centered on aging, anti-aging, oxidative stress, inflammation, and autophagy, underscoring the mechanistic and therapeutic research trajectories shaping our understanding of healthspan, senescence, and mental health within aging populations. Our findings suggest that the integration of circadian biology into aging and anxiety research has gained traction, particularly in addressing chronic comorbidities and the molecular underpinnings of age-related dysfunction. To our knowledge, this is the first bibliometric study to systematically explore the interdisciplinary connections among circadian rhythm, anxiety disorders, comorbidity, and aging within the context of anti-aging research.

The network structure revealed not only distinct thematic silos such as circadian, anxiety, and aging comorbidity clusters but also critical conceptual gaps between them. This fragmentation suggests that the scientific literature has yet to fully integrate biological rhythm research with geriatric mental health and multimorbidity frameworks. By mapping these disconnections, the present analysis highlights where interdisciplinary links are missing and where future synthesis may yield the greatest scientific value. The visualized trends indicate a growing need for conceptual models that unify circadian regulation, stress responses, and age-related vulnerability. Accordingly, future research should prioritize cross-domain collaborations and mechanistic studies capable of bridging these silos, thereby informing more comprehensive risk models and translational strategies for aging populations.

## Data Availability

The datasets generated and/or analyzed during the current study are available from the corresponding author upon reasonable request.
